# Effectiveness of the heartbeat interval error and compensation method on heart rate variability analysis

**DOI:** 10.1049/htl2.12023

**Published:** 2022-03-08

**Authors:** Ayaka Shintomi, Shintaro Izumi, Masahiko Yoshimoto, Hiroshi Kawaguchi

**Affiliations:** ^1^ Graduate School of System Informatics Kobe University 1‐1 Rokkodai‐cho Nada‐ku Kobe Hyogo Japan

## Abstract

The purpose of this study is to evaluate the effectiveness of heartbeat error and compensation methods on heart rate variability (HRV) with mobile and wearable sensor devices. The HRV analysis extracts multiple indices related to the heart and autonomic nervous system from beat‐to‐beat intervals. These HRV analysis indices are affected by the heartbeat interval mismatch, which is caused by sampling error from measurement hardware and inherent errors from the state of human body. Although the sampling rate reduction is a common method to reduce power consumption on wearable devices, it degrades the accuracy of the heartbeat interval. Furthermore, wearable devices often use photoplethysmography (PPG) instead of electrocardiogram (ECG) to measure heart rate. However, there are inherent errors between PPG and ECG, because the PPG is affected by blood pressure fluctuations, vascular stiffness, and body movements. This paper evaluates the impact of these errors on HRV analysis using dataset including both ECG and PPG from 28 subjects. The evaluation results showed that the error compensation method improved the accuracy of HRV analysis in time domain, frequency domain and non‐linear analysis. Furthermore, the error compensation by the algorithm was found to be effective for both PPG and ECG.

## 1 INTRODUCTION

In recent years, the development of biological signal measurement technology has enabled the use of wearable sensors for the constant monitoring of biological signals in daily life. In this study, we focus on the measurement of the heart rate and heart rate variability (HRV) analysis using wearable sensors. Ischemic heart disease and stroke are the leading causes of death worldwide, and deaths due to diabetes and dementia are increasing. Early detection and prevention of these diseases are essential to improve the quality of life, which may be achieved through the constant monitoring of biological signals using wearable sensors.

HRV analysis [1] is a method used to predict cardiac diseases [2] and estimate autonomic nervous system activity by monitoring heartbeat intervals [3]. The heartbeat constantly fluctuates due to the activity of the autonomic nervous system, by analysing the characteristics of the heartbeat, the stress state can be estimated [4]. In the literature [5], a correlation between stress and cardiovascular disease has been reported. It has also been suggested that there exists a relationship between the autonomic nervous system and cognitive function [6, 7].

In general, an electrocardiogram (ECG) is used for HRV analysis. However, owing to the inconvenience of measuring ECG in daily life, photoplethysmography (PPG) are more widely used than ECG in heart rate measurement using wearable sensors [8]. An ECG measures the potential difference on the body surface attributable to the electrical activity of the heart. The PPG sensors irradiate green or red light on the body surface and measure the reflected wave with a photodiode. The pulse interval highly correlated with heartbeat interval is obtained by detecting the peak from the PPG signals. PPG sensors use LEDs, which have the problem of high‐power consumption. Therefore, the sampling rate of PPG is often reduced in wearable sensors to reduce power consumption [9, 10, 11]. However, when the sampling rate is low, the effect of sampling error cannot be ignored.

Moreover, the pulse interval extracted from PPG includes errors due to blood pressure, body position, blood flow velocity, and stiffness of the vessel wall. Previous studies [12, 13, 14] have demonstrated that the effect of interval errors on HRV analysis when using pulse intervals obtained from PPG is limited, and the error using PPG is negligible when the patient is at rest. Reference [15] evaluate HRV indices using PPG with 29 hospitalized patients and the paper pointed out that some of the indices affected by the high‐frequency content of the HRV. These studies have been evaluated using data with a sampling rate of 125 Hz or higher. In the literature [16], it has been stated that the sampling rate of ECG for HRV analysis should be 128 Hz or higher. However, the impact of errors at low sampling rates on the analysis has not been quantitatively evaluated.

**FIGURE 1:**
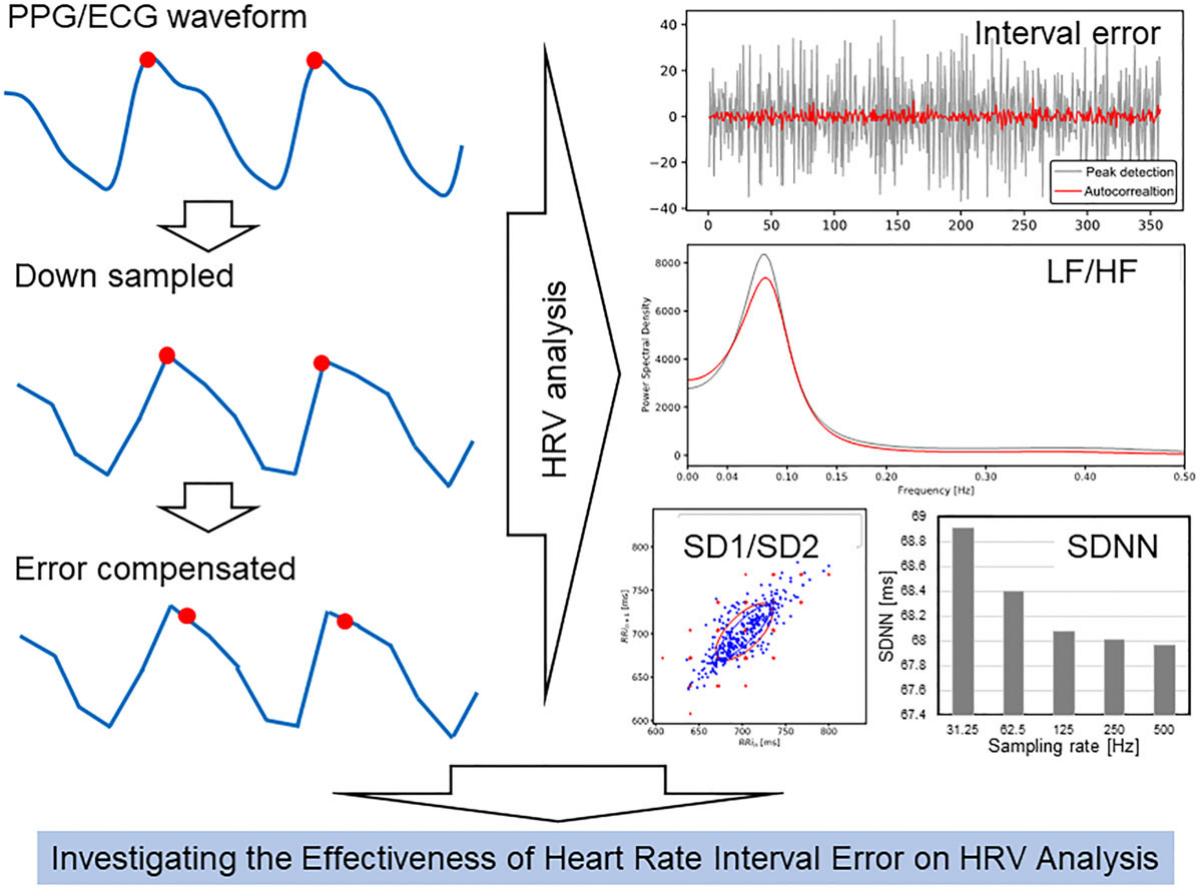
Concept image of this study

Figure 1 illustrates the concept of this paper. Our objective is to quantify the effect of heartbeat interval error on HRV analysis. In this paper, we used a data set in which ECG and PPG were measured simultaneously, and evaluated the effect of the sampling rate of PPG. We also evaluated the effect on HRV analysis when a compensation algorithm [17, 18] is used to compensate the errors caused by the peak detection.

The rest of this paper is organized as follows: In Section 2, we discuss HRV analysis and its evaluation indices. In Section 3, the effect of heartbeat interval error on HRV analysis and related research on sampling error compensation are presented. Section 4 describes the analysis method, and Section 5 presents the evaluation results. Section 6 discusses the results, and Section 7 concludes the paper.

## 2 HEART RATE VARIABILITY ANALYSIS

HRV analysis is a method used for detecting cardiac diseases and evaluating the autonomic nervous system activity by extracting indices from the heart rate variability. Frequency‐domain, time‐domain and non‐linear indices are used in HRV analysis. The details of those indices are described below.

### 2.1 Frequency‐domain indices

Frequency analysis was performed on the heartbeat intervals of the time series, and the spectral intensity was used as an index. First, heartbeat interval was resampled at 1 Hz by using spline interpolation. Then, the power spectral density (PSD) was calculated by Welch's method. The frequency‐domain index is LF/HF, where LF represents the power in the low‐frequency range (0.04–0.15 Hz) of R to R interval (RRI) from ECG or pulse to pulse interval (PPI) from PPG and is said to reflect the sympathetic and parasympathetic activity. HF represents the power in the high‐frequency range (0.15–0.4 Hz) of RRI or PPI and is said to reflect the parasympathetic activity. LF/HF is the ratio between LF and HF, indicating a balance between the sympathetic and parasympathetic activities.

### 2.2 Time‐domain and non‐linear indices

SDNN is index calculated in the time domain using time‐series heartbeat intervals. SDNN denotes the standard deviation of the heartbeat interval, and is an index that reflects the effects of both short‐term and long‐term variations in the heartbeat [19].

Poincaré plot is a non‐linear method, and SD1, SD2, and SD1/SD2 are non‐linear indices. SD1 and SD2 denote the crosswise and lengthwise standard deviations of the Poincaré plot of the heartbeat interval, respectively, and SD1/SD2 represents their ratio. SD1 and SD2 reflect the short‐term and long‐term heart rate variabilities, respectively [19]. Therefore, SD1/SD2 is an indicator of the balance between the short‐term and long‐term variabilities.

## 3 RELATED WORK

As mentioned above, RRI is generally used for HRV analysis. Recently, PPI is also widely used to obtain heart rate in wearable sensors instead of RRI, because it is easier to measure in daily life. However, Reference [15] states that the PPI increases relative error of some of the indices such as LF/HF and SD1/SD2. Reference [20] also shows that the use of PPI increases the error of HF and SD1.

PPI inherently contains errors compared to RRI. Several previous studies have demonstrated that ECG and PPG provide similar information about the autonomic nervous system. According to references [21, 22], PPG data measured using wearable devices are susceptible to motion artifacts, and reducing the effects of motion artifacts will allow for reliable HRV analysis. Reference [23] applies adaptive noise cancellation based on recursive least square using the Lambert‐Beer law and the hue‐saturation‐intensity model to reduce motion artifacts in PPG waveforms obtained by non‐contact sensors. Reference [24] also states that the pulse width and amplitude of PPG can provide accurate information about the autonomic nervous system. Furthermore, reference [25] states that HRV analysis using PPG can be used as a surrogate for that using ECG even under high pressure.

**FIGURE 2:**
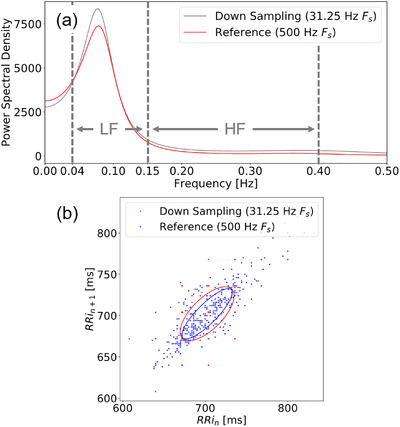
(a) Example of frequency domain HRV analysis at low sampling rate (31.25 Hz) and high sampling rate (500 Hz) ECG, (b) examples of Poincaré plot at low sampling rate (31.25 Hz) and high sampling rate (500 Hz) ECG

One of the limitations of heart rate measurement using wearable sensors is that the sampling rate has to be reduced to achieve low‐power consumption. This is especially important for LED‐based PPG. Figures 2a and 2b show calculated LF/HF and Poincaré plot from low sampling rate (31.25 Hz) and high sampling rate (500 Hz) ECG from healthy subject at rest, respectively. These results indicate that the HRV analysis results are affected by the sampling error.

## 4 METHOD

In this section, we describe the dataset used for the evaluation and data pre‐processing methods.

### 4.1 Dataset

The Vortal dataset was collected from 39 healthy subjects [26]. The mean age of the subjects was 29 years (26–32 years). In this paper, we used data of 28 subjects from the Vortal dataset to evaluate each index. The data of the remaining 11 subjects were excluded because the noise level is too high to extract the RRI and PPI for more than 256 consecutive seconds.

In the Vortal dataset, the PPG signal of each subject was measured simultaneously using the ECG signal as a reference. The signal was sampled at 500 Hz. Each subject performed two 10‐minute measurements in the supine position; after the first measurement, subjects were asked to run on a treadmill, and the second measurement was performed immediately after the exercise. In this study, we used the data from the second measurement, which contained a wider range of RRI than the first measurement.

### 4.2 Heartbeat interval error compensation method

**FIGURE 3:**
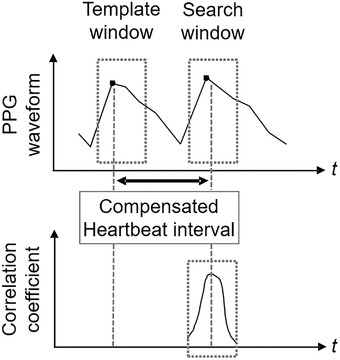
Overview of heartbeat error compensation using autocorrelation

To reduce the effect of sampling error, several error compensation algorithms have been proposed. In our previous work [17], we proposed a heartbeat interval extraction method based on linear interpolation and autocorrelation to reduce the sampling error. Figure 3 shows an overview of the error compensation method using autocorrelation. Since ECG waveforms are quite similar in a short period of time, the autocorrelation using the similarity of P, Q, R, S, and T waves can extract the heartbeat interval with higher accuracy than the conventional method that simply detects the peak of R wave. another study [18], the similarity around the peak was improved by adding filter processing to the time series signal before autocorrelation and using a two‐line approximation.

These methods were proposed to improve the accuracy in pulse interval error and frequency domain indices. Note that only two types of indices were used for evaluation in each study, and time domain indices and indices obtained by non‐linear analysis were not evaluated.

### 4.3 Preprocessing for HRV analysis

**FIGURE 4:**
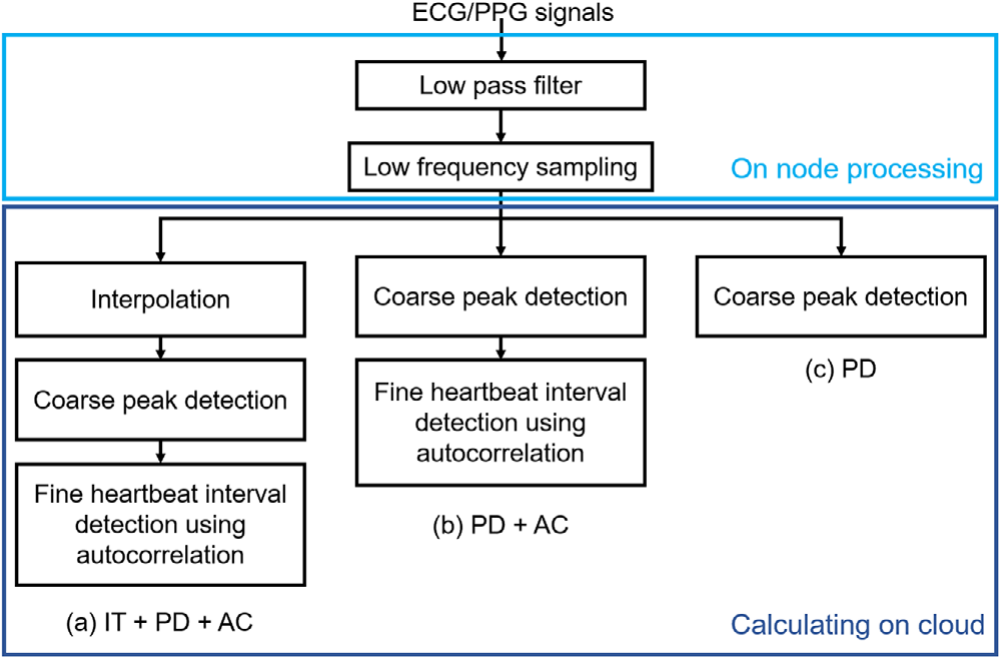
Flowchart of heartbeat interval evaluation (PD: peak detection, IT: interpolation, AC: autocorrelation)

Figure 4 illustrates a flowchart of the preprocessing method for the ECG and PPG waveforms. To improve the energy efficiency of edge devices, on‐node processing uses lower sampling rate to reduce the amount of data. On the other hand, the server side performs heavy completion and correlation calculations to improve the accuracy. In this study, to evaluate the effect of sampling rate on HRV analysis, the ECG and PPG data measured at 500 Hz were down‐sampled to 250, 125, 62.5, 31.25 Hz, respectively. To generate down‐sampled data, we first applied a low‐pass filter with a cutoff frequency of half the desired sampling rate, and then down‐sampled the data. This process prevents aliasing noise and reduces the amount of computation in the cloud.

Furthermore, to evaluate the impact of error compensation methods on the HRV analysis results, we used the three methods depicted in the flowchart in Figure 4, namely a method combining linear interpolation, peak detection and autocorrelation ((a) IT+PD+AC), a method combining peak detection and autocorrelation ((b) PD+AC), and a method using only peak detection ((c) PD).

In the coarse peak detection, the R‐wave and PPG wave peak were detected from the down‐sampled waveforms in each method. The peaks were simply detected from the maximum value. In the case of linear interpolation, the down‐sampled data is up‐sampled again to 500 Hz using interpolation.

In the fine peak detection, the peak positions were corrected using the error correction method described in Section 3.2. A template window was created based on the first detected peak position. The window length is set adaptively, and the template window was set to 180–260 ms width around the peak of the R wave in ECG, and set to 90–170 ms width around the peak of the PPG waveform.

Next, a search window with the same width as the template window is set and the correlation coefficient is calculated around the next peak. The distance between the windows with the largest correlation coefficient indicates the corrected interval. The heartbeat and pulse intervals extracted by these methods were used.

## 5 RESULT

### 5.1 Pulse interval error

First, we evaluated the root mean square error (*RMSE*) (1) to assess the difference between the *PPI* obtained from PPG and the *RRI* obtained from ECG.

(1) $$\begin{array}{c} RMSE=\sqrt{\frac{1}{n}\sum_{k=1}^{n}{\left(PPI_{k}-RRI_{k}\right)}^{2}} \end{array}$$

where $RRI_{k}$ is the *k*th interval obtained from ECG at 500 Hz, and $PPI_{k}$ is the *k*th interval obtained from PPG; n is the number of data points.

**FIGURE 5:**
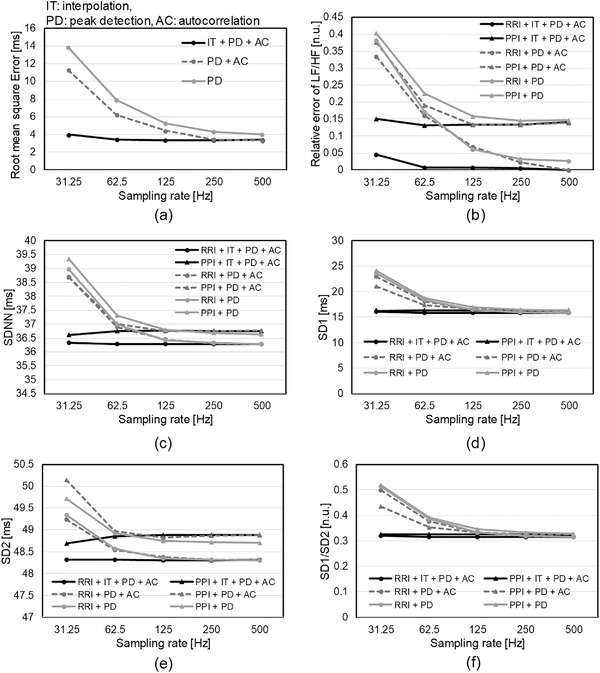
HRV analysis results using Vortal dataset; (a) root mean square error of heartbeat interval, (b) relative error of LF/HF, (c) SDNN, (d) SD1, (e) SD2, and (f) SD1/SD2

Figure 5a shows the average RMSE for all subjects at each sampling rate. The IT+PD+AC method demonstrates the least variation with the sampling rate and the best accuracy of pulse interval calculation results. The comparison of the results obtained using PD+AC and PD reveals that the error is small when using PD+AC at any sampling rate. The difference between the results obtained using PD+AC and PD tends to increase slightly as the sampling rate decreases.

### 5.2 Effect of sampling rate on frequency‐domain indices

LF/HF was evaluated using the relative error shown in (2).

(2) $$\begin{array}{c} Relative\;error=\frac{\left|average_{ref}-average_{f_{s}}\right|}{average_{ref}} \end{array}$$

where $average_{ref}$ is the average value of LF/HF of the reference calculated from *RRI* at 500 Hz, and $average_{f_{s}}$ is the LF/HF calculated from *RRI* or *PPI* at a sampling rate of *fs*.

Figure 5b depicts the evaluation results of the LF/HF relative error at each sampling rate, and similar to the *RMSE* results, the variation of IT+PD+AC with the sampling rate is not significant. In addition, when *PPI* is used to calculate LF/HF, the relative error is higher at almost all sampling rates than when *RRI* is used. However, the comparison between the results obtained using PD+AC and PD indicates that the difference in the relative error between the case using *RRI* and that using *PPI* decreases as the sampling rate decreases.

### 5.3 Effect of sampling rate on time‐domain and non‐linear indices

Figure 5c–f depicts the evaluation results for SDNN, SD1, SD2, and SD1/SD2, respectively. Note that the time‐domain and non‐linear indices were evaluated based on the value of each index.

The comparison of SD1 and SD2, depicted in Figures 5d and 5e, shows that the variation in SD1 is larger when the sampling rate is varied than SD2. The absolute value of the difference between SD1 calculated from RRI at 31.25 Hz and that at 500 Hz is 7.2 ms, and the absolute value of the difference with regard to SD2 is 0.9 ms. The peak detection method is PD+AC. The evaluation results for SDNN, SD1, and SD1/SD2 shown in Figure 5c, 5d, and 5f, respectively, show the same trend. The error by using PPI is limited, but the error increases at lower sampling rates only using PD. The total error is improved by adding the AC method, and the sampling error is also corrected by adding IT. However, when SD2 is calculated using the PPI extracted from the 31.25 Hz PPG, the results are closer to the reference value when PD is used than when PD+AC is used.

## 6 DISCUSSION

In this study, we quantitatively evaluated the effects of heartbeat interval error and error compensation on each index of HRV analysis. The evaluation results show that each index can be improved using the error compensation algorithm.

A previous study evaluated the effect of a reduced sampling rate on the analysis results when using PPI for HRV analysis, and concluded that in situations wherein a perfect PPG peak can be detected, a sampling rate of 25 Hz may be as reliable as that obtained from ECG [27]. This study demonstrated that error compensation can reduce the relative error of LF/HF to 8.6% when PPG with a sampling rate of 31.25 Hz is used for HRV analysis, in comparison with a relative error of 16.6% obtained without error compensation.

The evaluation results shown in Figure 5 indicate that the intrinsic error caused by using PPI appears to be larger for LF/HF than for SDNN. In addition, the relative error of SD1 is larger than SD2. These results support the results obtained in the literatures [15, 20].

SD1 and SDNN results in Figure 5 show that the PPI has higher accuracy in these indices when the sampling rate is less than 62.5 Hz. This slight counterintuitive result is caused by the fact that ECG waveforms are more susceptible to sampling errors than PPG, because the ECG waveform has higher frequency components compared to PPG.

In addition, reference [28] discusses the difference in HRV analysis accuracy based on the indices. The study concludes that a sampling rate of 100 Hz for time‐domain indices and 250 Hz for frequency‐domain indices is necessary when performing HRV analysis using ECG. The subjects in this experiment were limited to those with acute poisoning. In Section 5, error compensation using autocorrelation is shown to be effective for indices other than SD2. It is reasonable that the difference in the effectiveness of autocorrelation appears to vary based on the indices.

The data length of the heartbeat interval used for HRV analysis affects the accuracy of the analysis results. Previous study [29] claims that the use of data longer than one minute did not have a significant effect on HRV analysis, but the relative error increased when the data length was less than one minute. Therefore, 256‐s sequence of data was used in this study.

## 7 CONCLUSION

In this study, we evaluated the effectiveness of the heartbeat interval error compensation algorithm. ECG and PPG datasets measured in real environments were used in the evaluation. The results showed that the error compensation algorithm was effective for five of the six indices used in this study, except for SD2. HRV analysis could be performed with high accuracy even at a low sampling rate of 31.25 Hz when using linear interpolation and autocorrelation for peak detection. Furthermore, the analysis results indicate that PPI and RRI still have several milliseconds inherent errors even after compensation. The impact of this error on the application of HRV analysis needs to be investigated in the future work.

## CONFLICT OF INTEREST

No.

## FUNDING INFORMATION

No.

## Data Availability

The data that support the findings of this study are available from King's College London. Restrictions apply to the availability of these data, which were used under license for this study.
